# A Day Awake Attenuates Motor Learning-Induced Increases in Corticomotor Excitability

**DOI:** 10.3389/fnhum.2016.00138

**Published:** 2016-03-30

**Authors:** Toon T. de Beukelaar, Jago Van Soom, Reto Huber, Nicole Wenderoth

**Affiliations:** ^1^Movement Control and Neuroplasticity Research Group, Department of Kinesiology, Katholieke Universiteit LeuvenLeuven, Belgium; ^2^Child Development Center and Pediatric Sleep Disorders Center, University Children’s Hospital ZurichZurich, Switzerland; ^3^Neural Control of Movement Laboratory, Department of Health Sciences and Technology, Eidgenössische Technische Hochschule Zurich (ETH Zürich)Zurich, Switzerland

**Keywords:** synaptic homeostasis hypothesis, transcranial magnetic stimulation, finger sequence tapping, motor learning, sleep

## Abstract

The “synaptic homeostasis hypothesis” proposes that the brain’s capacity to exhibit synaptic plasticity is reduced during the day but restores when sleeping. While this prediction has been confirmed for declarative memories, it is currently unknown whether it is also the case for motor memories. We quantified practice-induced changes in corticomotor excitability in response to repetitive motor sequence training as an indirect marker of synaptic plasticity in the primary motor cortex (M1). Subjects either practiced a motor sequence in the morning and a new motor sequence in the evening, i.e., after a 12 h period of wakefulness (*wake group*); or they practiced a sequence in the evening and a new sequence in the morning, i.e., after a 12 h period including sleep (*sleep group*). In both wake and sleep groups motor training improved movement performance irrespective of the time of day. Learning a new sequence in the morning triggered a clear increase in corticomotor excitability suggesting that motor training triggered synaptic adaptation in the M1 that was absent when a new sequence was learned in the evening. Thus, the magnitude of the practice-induced increase in corticomotor excitability was significantly influenced by time of day while the magnitude of motor performance improvements were not. These results suggest that the motor cortex’s potential to efficiently adapt to the environment by quickly adjusting synaptic strength in an activity-dependent manner is higher in the morning than in the evening.

## Introduction

The synaptic homeostasis hypothesis (Tononi and Cirelli, 2003, 2006) assumes that a net increase in synaptic strength occurs when awake due to long-term potentiation (LTP) triggered by learning (Muellbacher et al., 2002; Silva, 2003; Rosenkranz et al., 2007a) or due to synaptic plasticity reflecting statistical regularities of the environment experienced during wakefulness (Cirelli and Tononi, 2000; Tononi and Cirelli, 2001, 2003; Huber et al., 2013). This increase in synaptic strength is believed to reduce neuronal selectivity, i.e., firing in response to a specific stimulus, but also limits the capacity to undergo further synaptic plasticity (saturation of learning capabilities; Tononi and Cirelli, 2003, 2006, 2014). The synaptic homeostasis hypothesis predicts that sleep “downscales” or renormalizes the overall synaptic strength hereby improving signal-to-noise ratio and restoring the brain’s energy balance and cellular homeostasis (Tononi and Cirelli, 2014). Using a plausible computational model of sleep-dependent renormalization, it has been predicted that the human brain’s ability to form new memories is hereby renormalized in the morning following sleep (Olcese et al., 2010). In accordance with this latter prediction, behavioral studies testing the formation of declarative memories showed that sleep was beneficial for memory consolidation (Born et al., 2006; Gais et al., 2006) and that learning capacity was higher in the morning (i.e., after 12 h including sleep) than in the evening (i.e., after 12 h without sleep; Kvint et al., 2011). Moreover, sleep deprivation caused a substantial impairment in learning capacity (McDermott et al., 2003; Yoo et al., 2007; Mander et al., 2011). By contrast, for motor learning, and most notably for sequence learning, it has been shown that while sleep is beneficial for consolidation and retention performance, particularly when performance saturation was reached during prior training (Kvint et al., 2011), behavioral measurements of sequence learning capacity did not differ in the morning compared to the evening (Fischer et al., 2002; Walker et al., 2002; Brawn et al., 2010; Kvint et al., 2011; Sale et al., 2013).

Here, we test the prediction that motor learning-induced synaptic plasticity is attenuated after a period of wakefulness. Transcranial magnetic stimulation (TMS) was used to estimate a person’s capacity to undergo synaptic plasticity in the primary motor cortex (M1) either after 12 h of wakefulness or after the same period including sleep. Synaptic plasticity was probed in response to repetitive training of a five-element motor sequence which has been shown to modify the functional organization of the motor system, a phenomenon known as use-dependent plasticity (Classen et al., 1998; Muellbacher et al., 2002; Ziemann et al., 2004; Rosenkranz and Rothwell, 2006; Stefan et al., 2006; Perez et al., 2007; Rosenkranz et al., 2007a,b; Huang et al., 2011; Zhang et al., 2011; Bisio et al., 2015).

In humans, use-dependent plasticity within M1 is indicated by larger motor-evoked potentials (MEP) after training than at baseline (Classen et al., 1998; Perez et al., 2007; Rosenkranz et al., 2007a,b). This increase in corticomotor excitability most likely results from training-induced synaptic plasticity leading to strengthening of intracortical neuronal ensembles (Rioult-Pedotti et al., 1998, 2000; Bütefisch et al., 2000), it is *N*-Methyl-D-aspartate (NMDA) receptor-dependent and it is strongly reduced by γ-aminobutyric acid type A (GABA_A_) receptor mediated inhibition (Bütefisch et al., 2000). Moreover, use-dependent plasticity occluded subsequent induction of LTP via paired-associative stimulation (PAS) protocols in accordance to principles of homeostatic metaplasticity as predicted by the Bienenstock-Cooper-Munro theory (Kirkwood et al., 1996; Stefan et al., 2006; Rosenkranz et al., 2007a), thus suggesting that this type of learning saturates synaptic plasticity. Together, these findings strongly suggest that use-dependent plasticity activates LTP-like mechanisms in humans (Bütefisch et al., 2000; Stefan et al., 2006; Rosenkranz et al., 2007a) which are reflected by changes in corticomotor excitability.

The synaptic homeostasis hypothesis predicts that the brain’s capacity to undergo synaptic plasticity is reduced after a prolonged period awake, while this ability is restored after a night of sleep. In line with this theory, we hypothesize that inducing use-dependent plasticity in the morning by practicing one motor sequence will result in larger increases in corticomotor excitability than practicing a new motor sequence in the evening because overall synaptic strengthening during the waking day will diminish the potential to further increase synaptic efficiency.

## Materials and Methods

### Subjects

Nineteen naïve (no musicians, no prior experience with the task), healthy, right-handed (Oldfield, 1971) subjects (1 female, mean ± SD age; 21.9 ± 1.1 years) participated in this experiment. All subjects signed a written informed consent prior to participation and were screened for adverse reactions to TMS when they complied with the inclusion criteria. The experimental procedure was approved by the local Ethics Committee for Biomedical Research at the Catholic University of Leuven in accordance to The Code of Ethics of the World Medical Association (Helsinki, 1964).

### General Setup

Participants were seated in a comfortable chair with their right forearm resting in a neutral position and performed the behavioral task on a laptop positioned in front of them (for details see below). Subjects wore a tight fitting swimming cap which allowed to outline the TMS coil position and helped placing the TMS coil appropriately in each session.

### Electromyographic Recordings (EMG) and TMS

EMG recordings and TMS acquisition were performed in accordance to a standard protocol described in Alaerts et al. (2011). Focal TMS was applied with a 70 mm figure-of-eight coil connected to a Magstim 200 stimulator (Magstim, Whitland, Dyfed, UK). The coil was positioned over M1 of the left hemisphere, tangential to the scalp with the handle pointing backwards and laterally at 45° away from the mid-sagittal line (Pascual-Leone et al., 2002). The optimal scalp position (“hotspot”) for stimulating the right first dorsal interosseous (FDI) and its rest motor threshold (rMT; lowest stimulus intensity evoking MEPs with an amplitude of at least 50 μV in 5 out of 10 consecutive stimuli) were determined (Rossini et al., 1994; Table 1).

**Table 1:** **Subject data**.

			rMT (%)		Hotspot	
	Age (yrs)	Oldfield (%)	Ses 1	Ses2	Ses 1	Ses 2	Sleep (h)	Sleep quality (0–10)
Wake	21.8 ± 1.2	81.7 ± 17.0	36.1 ± 3.9	35.8 ± 3.1	*x*: 5.3 ± 1.0	*x*: 5.2 ± 0.8	7.2 ± 1.0	7.5 ± 1.4
(*n* = 9)					*y*: 0.8 ± 0.4	*y*: 0.4 ± 0.5		
Sleep	21.9 ± 1.1	90.5 ± 12.6	37.1 ± 5.4	37.3 ± 5.5	*x*: 4.8 ± 0.9	*x*: 4.8 ± 0.9	8.1 ± 1.9	6.7 ± 1.9
(*n* = 10)					*y*: 0.7 ± 0.8	*y*: 0.8 ± 0.9	7.1 ± 0.8	6.6 ± 1.5

*Groups were matched regarding age and gender (n = 19; 1 female). The rest motor threshold (rMT) indicates the lowest stimulus intensity evoking MEPs with amplitudes of at least 50 μV in 5 out of 10 consecutive stimuli. Hotspot location is reported as the distance in cm relative to the vertex. The table shows x, y coordinates with x being the lateral-medial distance (positive values are located left to the nasion-inion line) and y the anterior-posterior distance (positive values are located anterior to the vertex). Subjects were asked about their hours of sleep and sleep quality (i.e., score between 0–10) before the practice sessions. The wake group was asked about the night before the morning session and the sleep group about both the night before the evening as the next morning session. No differences were observed considering rMT, hotspot location or hours of sleep between groups (t ≤ 0.73; p ≥ 0.48) and between sessions (t < 0.76; p > 0.47). Data are represented as mean ± SD*.

Disposable Ag-AgCl surface electrodes (Blue sensor SP Surface) were used to record EMG from the FDI. The first electrode was placed on the belly, the second on the tendon of the muscle and a third on a bony prominence (reference electrode). The signals were sampled at 5000 Hz (CED Power 1401, Cambridge Electronic Design, UK), amplified, band-pass filtered (5–1000 Hz), and stored on a PC for offline analysis. Pre-stimulus EMG recordings were used to assess the presence of unwanted background EMG activity in the 110–10 ms time interval preceding the magnetic pulse.

Corticomotor excitability was quantified by measuring input-output curves (IO curve) using 90, 115, 140, 165 and 190% of rMT. One IO curve consisted of 20 MEPs per intensity. They were acquired in two blocks of 50 MEPs so that per block 10 stimulations were acquired for each of the five intensities. In between blocks a rest period of approximately 2 min was provided. Within one block, the interstimulation interval ranged from 5–9 s resulting in a total block time of 6 min 30 s.

### Behavioral Task

**Figure 1:**
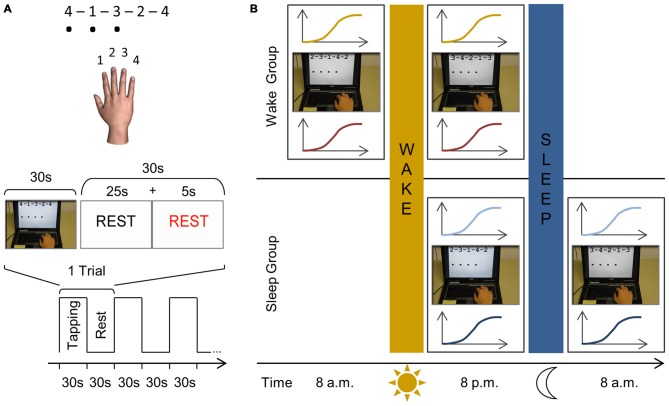
**Schematic representation of the finger sequencing task and experimental protocol. (A)** The motor task is performed with the right dominant hand on a laptop keyboard. Three different five-element sequences are used throughout the experiment, each consisting of four numeric keys (A: 4–1–3–2–4; B: 2–3–1–4–2; C: 3–4–2–1–3). Each number represented a finger; with “1” being the index finger, “2” the middle finger, etc. The sequence to be executed (e.g., sequence A) was shown on the computer screen using the same numbering system to reduce the likelihood of the task including a working memory component. While performing the task, a black dot appeared on the screen indicating that a key had been pressed. Key presses were recorded and no feedback was provided regarding task accuracy. An experimental trial consisted of 30 s of sequence tapping followed by a rest period of 30 s to prevent fatigue. During the practice sessions, this experimental trial is repeated 12 times so that subjects are mass trained for 12 min in total. Participants were instructed to type the sequences as quickly and as accurately as possible and were motivated continuously throughout the experiment. **(B)** In both groups, we first determined the rest motor threshold (rMT) and hotspot for each subject. In the wake group, subjects are first tested at 8 a.m. and IO curves are measured before (yellow) and after training (red) the finger sequencing task for 12 min (e.g., sequence B). At 8 p.m., following a normal day, subjects are tested in an identical manner as during the morning session, however they are trained on a different sequence (e.g., sequence C). In the sleep group the first session takes place at 8 p.m. when IO curves are measured before (light blue) and after (dark blue) they practiced the finger sequencing task (e.g., sequence B). After a night’s sleep, they are tested again using the same procedure at 8 a.m. yet practicing a novel sequence (e.g., sequence C).

Subjects performed a computerized sequence tapping task (presented with E-Prime; Psychology Software Tools, Inc. Sharpsburg, PA, USA) adapted from Karni et al. (1998). The sequence to be executed was depicted on top of the laptop screen using a numbering system, with 1, 2, 3, and 4 corresponding to the index, middle, ring and little fingers of the right hand respectively. Throughout the experiment three different yet equally difficult sequences were used (A: 4–1–3–2–4; B: 2–3–1–4–2; C: 3–4–2–1–3). While tapping the sequence a black dot appeared on the screen below the current number every time the subject pressed a key indicating that a response was recorded without giving any accuracy feedback (Figure 1A). When a sequence was completed, the screen was refreshed so that the same sequence appeared on top without any black dots present. One experimental trial consisted of typing the given sequence for 30 s as many times as possible followed by a rest period of 30 s to prevent fatigue.

### Experimental Protocol

Subjects participated in a familiarization session, first practice session and second practice session. Each session required the acquisition of a new motor sequence (i.e., A, B or C with the order randomized across participants) which was repeatedly practiced within that session. During the familiarization session TMS was used to determine the FDI hotspot and rMT. Afterwards three experimental trials were performed (i.e., 30 s tapping of e.g., sequence A, followed by 30 s rest) which lasted 3 min in total. Subjects were then randomly assigned to one of two experimental groups.

The first experimental group, the *wake group*, started their first session at 8 a.m. (Figure 1B). The FDI hotspot and rMT were determined and corticomotor excitability was measured in the form of an IO curve. Subjects then performed motor training, i.e., they practiced a new motor sequence (e.g., sequence B) for 12 experimental trials (i.e., 30 s tapping followed by 30 s rest) which lasted 12 min in total. Subjects then left the lab and followed their typical daily routine and returned for their second session at 8 p.m., which followed the identical procedure but, importantly, a new motor sequence was acquired (e.g., C).

A similar procedure was followed in the second experimental group, the *sleep group*, but the first practice session took place in the evening at 8 p.m. After this first session subjects went home for a night of sleep and returned to the lab at 8 a.m. the following morning for the second session. The presentation of sequences A, B, and C was randomly assigned to familiarization, session 1 and session 2, and differed across subjects.

Before and during the testing day(s), subjects did not perform strenuous exercise, had no more than two cups of coffee a day and followed their normal sleep rhythm without taking additional naps during the day (as instructed and verified via self-report; Table 1).

### Data Analysis and Statistics

Key presses were recorded and accuracy (%) was calculated as the number of correct sequences divided by all completed sequences during each 30 s trial. Performance speed was measured as the time (s) between key presses, i.e., the inter-tap interval (ITI). A performance score was calculated for each subject and trial by dividing the accuracy percentage by the ITI, with higher scores indicating better performance (also see de Beukelaar et al., 2014). A repeated measures analysis of variance (ANOVA) was performed on performance scores with the between-subject factor *group* (wake, sleep) and the within-subject factors *session* (1st, 2nd) and *training block* (trial 1–12).

Corticomotor excitability was quantified by MEP peak-to-peak amplitude. MEP amplitude is known to be modulated by EMG background activation since slight voluntary contractions of the target muscle might increase MEP amplitude (Barker et al., 1986, 1987; Hess et al., 1987; Rothwell et al., 1987; Devanne et al., 1997; Nollet et al., 2003). Therefore pre-stimulus EMG recordings were used to assess the presence of unwanted background EMG activity in the 110–10 ms preceding the magnetic pulse and were quantified via root mean square scores (RMS) across this interval. For each subject and over all trials we calculated the mean and standard deviations of the background EMG so that values over + 2.5 standard deviation were removed from the analysis. Furthermore we considered MEP peak-to-peak amplitudes which exceeded Q3 + 1.5 × (Q3 & Q1) as outliers (3.1%) that were removed from further analysis, with Q1 denoting the first quartile and Q3 the third quartile computed over the whole set of trials for each subject. MEP amplitudes were averaged for each stimulation intensity of each IO curve that was recorded and these averages where then subjected to group statistics.

We first tested whether motor practice changed corticomotor excitability as quantified by the IO curve and whether these changes would differ between the first and second session. This analysis was performed separately for each experimental group using a repeated measures ANOVA (rmANOVA) with the within-subject factors *session* (1st, 2nd), *pre-post* (pre, post) and *intensity* (90, 115, 140, 165 and 190%). Next we tested whether baseline corticomotor excitability (i.e., measured prior to motor practice) changed from the first to the second session and calculated for each group a rmANOVA for the IO curve measured at pre, using the factors *session* (1st, 2nd) and *intensity* (90, 115, 140, 165 and 190%).

Finally we directly compared whether changes in corticomotor excitability induced by motor practice differed between sessions and groups. Therefore, we calculated the integral underneath the IO curve measured before and after motor practice (Carson et al., 2013), and calculated a facilitation index (FacInd) by:

$$\text{FacInd = }\int_{\text{Intensity1−5}}\text{MEPpost}/\;\int_{\text{Intensity1−5}}\text{MEPpre}$$

FacInd > 1 indicates that an increase in corticomotor excitability is observed from pre to post training, while a FacInd < 1 represents a decrease. The FacInds were calculated for the two sessions and the two groups and were entered into a repeated measures ANOVA with the between-subject factor *group* (wake, sleep) and within-subject factor *session* (1st, 2nd).

The alpha level for all statistical tests was set to 0.05 and significant interactions were further analyzed by the use of a Fisher’s LSD *post hoc* analysis. All statistical analyses were performed with Statistica 8 (StatSoft, OK, USA).

## Results

### Behavioral Results

**Figure 2:**
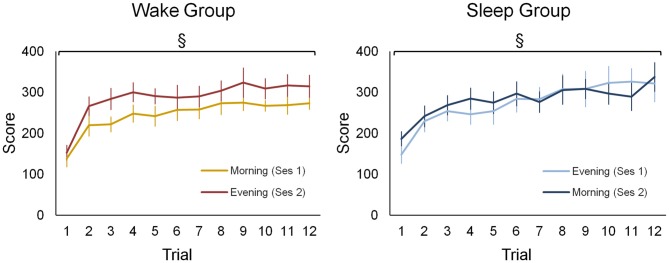
**Behavioral data of the wake and sleep group during both practice sessions.** For both groups and in each session a separate learning curve is shown for the sequence tapping task. There was a significant main effect for training block (*F*_(11,187)_ = 34.61; *p* < 0.001), yet no main effect for group or a higher interaction containing this group factor was found (*F* < 2.56; *p* > 0.12). A main effect for session (*F*_(1,17)_ = 5.01; *p* < 0.05) indicates that during the second session a higher performance is achieved in both groups. ^§^Represents a main training block effect (*p* < 0.001). Vertical bars indicate standard errors (SEs).

Both groups improved motor sequence performance during each of the practice sessions to a similar extent (Figure 2). Accordingly, statistics revealed a main effect for *training block* (*F*_(11,187)_ = 34.61; *p* < 0.001) but no main effect for *group* or a higher interaction containing the factor *group* (*F* < 2.56; *p* > 0.12). Additionally, performance was generally better in session 2 (310.52 ± 91.74) than in session 1 (297.97 ± 100.79) as indicated by a significant *session* main effect (*F*_(1,17)_ = 5.01; *p* < 0.05). However, there was no statistical evidence to suggest that learning gains were differential influenced be waking or sleeping since the *session × trial* interaction failed to reach significance (*F*_(11,187)_ = 0.78; *p* = 0.66).

### Neural Results

#### Wake Group

**Figure 3:**
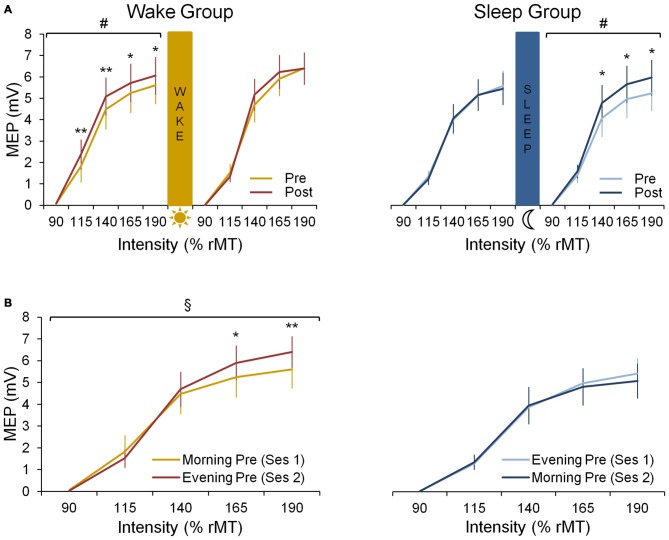
**Neural data. (A)** Input-0utput (IO) curves pre- and post-training in the first and the second practice session for both wake and sleep group. In the wake group (left panel) we show an increase in corticomotor excitability from pre-training (yellow curve) to post-training (red curve) in the morning session at 8 a.m. (*F*_(4,32)_ = 4.23; *p* < 0.01) but not in the evening session at 8 p.m. A Fisher LSD *post hoc* analysis shows that this effect in the morning is found for suprathreshold stimulation with intensities ≥ 115% rMT. In the sleep group (right panel) we show no increase in corticomotor excitability from pre-training (light blue curve) to post-training (dark blue curve) in their first session being the evening session at 8 p.m. During the second session on the consecutive morning at 8 a.m., a significant increase in excitability is seen from pre- to post-training (*F*_(4,36)_ = 2.68; *p* < 0.05), especially for supratreshold stimulation with intensities ≥ 140% rMT. **(B)** IO curves obtained pre-training in both sessions for both wake and sleep group. For the wake group an increase during the day is observed since the pre-training curve obtained in the evening session is increased compared to the pre curve in the morning (*F*_(4,32)_ = 5.96; *p* < 0.01), especially for supratreshold intensities ≥ 165% rMT. For the sleep group no difference between both pre curves is observed (*p* = 0.27). ^#^Indicates a prepost × intensity interaction effect (*p* < 0.05); ^§^Indicates a session × intensity interaction effect (*p* < 0.01); significant Fisher LSD *post hoc* analyses are represented by *p < 0.001 and **p < 0.0001. Vertical bars indicate SEs.

In the wake group, corticomotor excitability increased due to practice in the morning session while no such increase is seen in the evening session (Figure 3A). This is supported by a significant *session* × *prepost* × intensity interaction (*F*_(4,32)_ = 3.31; *p* < 0.05) and by follow up analyses revealing a significant prepost × intensity interaction (*F*_(4,32)_ = 4.23; *p* < 0.01) for the morning session, while significance was not reached in the evening session (*F*_(4,32)_ = 2.54; *p* = 0.06). This indicates that motor practice changed corticomotor excitability more strongly in the morning than in the evening.

When comparing the pre-training IO curves between the two experimental sessions, baseline excitability increased over a 12 h-day awake as indicated by a significant session × intensity interaction for the pre curves of both sessions (*F*_(4,32)_ = 5.96; *p* < 0.01; Figure 3B).

#### Sleep Group

In the sleep group, motor practice did not cause a significant increase of corticomotor excitability in the evening session (prepost × intensity interaction: *F*_(4,36)_ = 0.45; *p* = 0.77) while a significant increase was observed during the following morning session (*F*_(4,36)_ = 2.68; *p* < 0.05), i.e., after a night of sleep (Figure 3A). However, the session × prepost × intensity interaction did not reach significance most likely due to large inter-individual variability (*F*_(4,36)_ = 1.94; *p* = 0.13).

When investigating the evolution of the pre-training IO curves, we found no session × intensity interaction in the sleep group indicating that there was no significant change in baseline excitability overnight (*F*_(4,36)_ = 1.34; *p* = 0.27; Figure 3B).

#### FacInd

**Figure 4:**
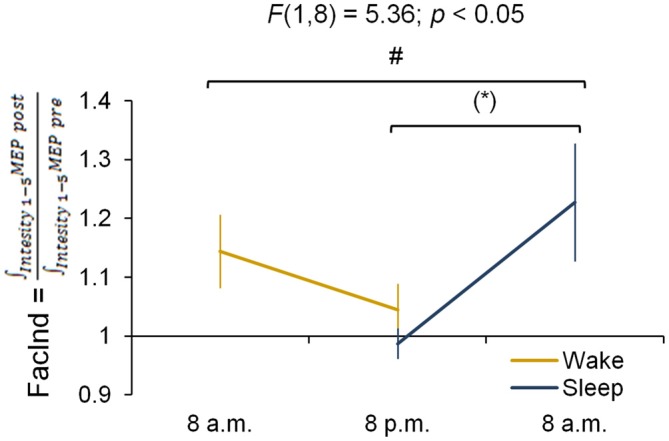
**Facilitation Index (FacInd).** The FacInd is calculated as a general measurement quantifying the potential to undergo learning-induced changes of corticomotor excitability. We divide the integrated motor-evoked potentials (MEP) amplitudes collected for each intensity after practice (i.e., area under the IO curve post-training) by the integrated MEP amplitudes of those collected before (i.e., area under the IO curve pre-training). This calculation shows that the potential to exhibit training-induced neural changes is dependent on time of day and is higher in the morning compared to the evening in both experimental groups *p = 0.052. ^#^Indicates a group × session interaction effect (*p* < 0.05); marginally significant Fisher LSD *post hoc* analysis is represented by *p = 0.052. Vertical bars indicate SEs.

The FacInd was calculated to directly test whether the potential to undergo changes in corticomotor excitability differed when practice sessions were either separated by 12 h awake (wake group) or 12 h including sleep (sleep group). Figure 4 shows that the FacInd of the wake group was higher in the morning (indicating that excitability changed by approximately 14.4 ± 18.6% in response to motor practice) than in the evening (excitability changes were only 4.4 ± 13.5%). The sleep group, by contrast, exhibited the opposite pattern with a lower FacInd in the evening (−1.3 ± 8.1%) than the next morning (22.7 ± 31.8%), i.e., after a night of sleep. Importantly, statistics revealed a significant group × session interaction (*F*_(1,8)_ = 5.36; *p* < 0.05) suggesting that wakefulness decreases the ability to change corticomotor excitability in response to motor practice whereas the ability to exhibit use-dependent neural changes was re-established at the next morning after a night of sleep.

## Discussion

In the present study, we tested the prediction that motor learning-induced synaptic plasticity is attenuated after a period of wakefulness. The capacity to undergo synaptic plasticity was probed by measuring changes in corticomotor excitability in response to acquiring a finger sequence tapping task, a learning paradigm that is well-known to induce use-dependent plasticity in M1. Our main finding is that the capacity to increase corticomotor excitability in response to motor practice (a marker of training-induced synaptic strengthening) is reduced after 12 h of wakefulness during the day.

### Behavioral Data

In this study, we used a sequence tapping task to induce use-dependent plasticity. The advantage of this task is that a similar learning process can be induced twice, a requirement of our experimental design. In order to account for potential differences in complexity, we randomized the presentation of sequences in a balanced order. Furthermore, a familiarization session was performed, so that subjects knew the general paradigm in order to minimize novelty effects.

Unlike the neural measurements, the behavioral data did not show differential performance gains in the morning compared to the evening sessions. It is important to note that task performance is not a “pure” measurement of memory formation because it is strongly influenced by fatigue, attention, alertness and motivation (Karni et al., 1998; Robertson et al., 2004a,b). For the sequence tapping task used here it is well-known that time of day does not result in differential motor learning gains (Fischer et al., 2002; Walker et al., 2003; Brawn et al., 2010), and that the beneficial effect of sleep has only been demonstrated for retention performance (i.e., an indirect marker of consolidation) but not for restoring motor learning capacity. Thus, the absence of behavioral differences between morning and evening sessions when estimating sequence learning is highly consistent with previous findings.

Furthermore, the lack of differential behavioral findings between morning and evening sessions could be explained by the overall simplicity of the sequence tapping task in combination with the relative short acquisition phase in relation to the total training time. More specifically, Walker et al. (2002) reported an overall performance increase for sequence tapping of 59.3% over 12 × 30 s training trials with the largest increase occurring during the first 3 training trials (38.8%). Therefore, shortening the training period (e.g., less and/or shorter training trials) to prevent subjects to reach a performance plateau by the end of training could potentially be a more sensitive procedure to reflect more subtle time of day effects on behavior. Note also that the behavioral performance measurements are likely to reflect different learning processes: initially, skill acquisition ensures that the sequence is correctly represented at the neural level and that it is fluently performed which might cause large gains early in learning. By contrast, in a later phase, repetitive practice of the sequence is likely to activate mechanisms related to use-dependent plasticity; i.e., neural changes that are induced by extensively repeating movements within a specified time window (Classen et al., 1998; Bütefisch et al., 2000; Stefan et al., 2006; Rosenkranz et al., 2007a). This might explain why memory specific neurophysiological processes are not always accurately reflected by behavioral changes (Urbain et al., 2013).

### Influence of Waking vs. Sleep on the Potential for Increasing Corticomotor Excitability in Response to Motor Training

We found that the potential for increasing corticomotor excitability, measured as the difference between IO curves recorded before and after motor practice (similar to Lotze et al., 2003; Perez et al., 2004; Jensen et al., 2005; Stefan et al., 2006; Rosenkranz et al., 2007a,b; Zhang et al., 2011), was higher in the morning than the evening. It is important to keep in mind that we infer synaptic plasticity based on changes of corticomotor excitability as measured by single pulse TMS. This assumption is based on a large body of evidence reporting a robust increase of corticomotor excitability in response to extensive motor practice inducing use-dependent plasticity (Classen et al., 1998; Perez et al., 2007; Rosenkranz et al., 2007a,b), or other plasticity inducing protocols using transcranial direct current stimulation (tDCS; Romero Lauro et al., 2014), PAS (Stefan et al., 2000; Ridding and Uy, 2003) or theta-burst stimulation (Jacobs et al., 2012). Importantly, this rise in corticomotor excitability after extensive motor training has been shown to be of cortical origin (rather than reflecting changes at e.g., the spinal level; Rioult-Pedotti et al., 1998, 2000; Bütefisch et al., 2000), is specific for motor learning rather than for motor performance (Rosenkranz et al., 2007a), and it is abolished when synaptic plasticity is reduced either by blocking NMDA receptors or by increasing GABAergic inhibition with pharmacological agents (Bütefisch et al., 2000). Although there is compelling evidence that learning a finger sequence tapping task typically results in increased corticomotor excitability early after training, this might not be the case for all motor tasks (e.g., Tunovic et al., 2014) reported a delayed increase in corticomotor excitability). An alternative approach to probe neuroplasticity of human M1 is to experimentally induced LTP (typically by a PAS_LTP_ protocol) after motor training has been performed. According to models of homeostatic metaplasticity, the effect of LTP-inducing PAS (i.e., PAS_LTP_) is either reduced or even reversed to long-term depression (LTD) depending on the extent to which synaptic plasticity has been induced by prior motor practice. Combining motor training and PAS protocols is an elegant approach to test synaptic plasticity, however, the efficiency of PAS_LTP_ has been shown to be dependent on corticosteroid levels which are typically lowest early in the morning. Accordingly, PAS_LTP_ effects have been shown to be significantly smaller in the morning than in the evening (Sale et al., 2008). In the context of our paradigm this represents a potential confound and could therefore not be applied. Note however, that only the response to PAS_LTP_ was influenced by corticosteroid levels whereas MEP amplitudes were comparable over the day. Moreover, we controlled other confounding factors like the background EMG across the pre and the post session excluding the possibility that excitability changes were caused by pre-contraction. Therefore, we argue that the increase in corticomotor excitability in response to a standardized practice protocol as quantified by the FacInd is a surrogate marker of a person’s ability to undergo neuroplastic changes at the synaptic level (see also Rosenkranz et al., 2007b). Under this assumption, our data suggest that a day awake decreases the potential to show neural changes due to motor learning.

Our data indicate that there is no causal link between practice-induced changes in corticomotor excitability and practice-induced changes of motor behavior (Bestmann and Krakauer, 2015). It has been suggested that there is no straightforward relationship between MEP size (i.e., IO curve) and behavioral output following learning (Muellbacher et al., 2000, 2001; McDonnell and Ridding, 2006; Bagce et al., 2013). From our data, it is apparent that behavior can improve significantly even though corticomotor excitability remains virtually unchanged (as observed after a day of wakefulness). These short-term changes in corticomotor excitability as obtained in the morning sessions appear to indicate that M1 underwent adaptive changes resulting in increased efficiency of the activated neural network (Bestmann and Krakauer, 2015). In other words, a change in corticomotor excitability is not essential to learn a novel motor task, however, in order to efficiently learn the task the neural system needs to adapt. However, we showed that this capacity to learn and to efficiently adapt to the changing world around us is attenuated after a day of wakefulness. This interpretation is in line with the predictions of the synaptic homeostasis hypothesis proposing that one central function of sleep is to downscale overall synaptic strength, thus maintaining the brain’s efficiency by ensuring that neurons fire sparsely but selectively for important inputs. In this manner energy consumption is maintained at a sustainable level, and most importantly for our study the ability to learn is restored (Yoo et al., 2007).

### Potentiation of Synaptic Strength During Wakefulness

Our findings also support the notion that synaptic strength is potentiated during the day (Tononi and Cirelli, 2003, 2006, 2014) since corticomotor excitability measured prior to motor training increased from the morning to the evening in the wake group consistent with previous findings in humans (Huber et al., 2013) and animal models (Vyazovskiy et al., 2008).

Contrary to the wake group, in the sleep group we found only a slight non-significant decrease in baseline corticomotor excitability after a night of sleep. One has to note, though, that the sleep group did not participate in extra motor training during the day and that the synapses of the muscular representation probed with TMS might not have been strongly potentiated prior to the evening motor training. This is a key difference to the wake group since these subjects were exposed to intensive motor training in the morning. Furthermore, TMS stimulates pyramidal neurons in layer 5 transsynaptically, i.e., via interneurons located in layer 2/3 (Di Lazzaro and Ziemann, 2013). Higher MEPs might not only result from synaptic strengthening occurring within M1 but also from potentiated inputs to these M1 interneurons in layer 2/3 deriving from other areas (Bestmann and Krakauer, 2015). One primary candidate area that might have been activated by the tapping task is the striatum which has been shown to be involved in sequence learning and has dense reciprocal connections with M1 (Doyon et al., 2003; Doyon and Benali, 2005). Other likely input areas to M1 that also undergo changes in response to motor practice are parieto-premotor networks (Doyon and Benali, 2005). Even though these areas outside of M1 contribute to all phases of sequence learning it has been suggested that the time course is slightly different: thus while M1 probably undergoes most prominent synaptic changes during and immediately after practice, the striatum is believed to become increasingly more important during memory consolidation, i.e., during the first minutes and hours after the training has finished (Shadmehr and Holcomb, 1997; Doyon and Ungerleider, 2002; Frankland and Bontempi, 2005; Censor et al., 2010). Consequently we propose that short-term changes of corticomotor excitability as observed when comparing pre to post-training measurements might be predominantly driven by fast neuroplastic changes (which certainly involve M1), while long-term changes in corticomotor excitability as observed when comparing baseline excitability between the morning and the evening test might additionally be influenced by slow neuroplastic changes that occurred during consolidation and potentially, also in areas outside of M1.

### Interpretational Issues

The present study was designed in light of the synaptic homeostasis hypothesis, i.e., whether motor learning capacity is reduced after a day awake but restored in the morning after a night of sleep. Our results are in line with this prediction; however, the present study design does not allow us to dissociate the influence of sleep from the influence of circadian rhythms. Indeed it has been shown that performance of certain motor tasks show time of day effects (Miller et al., 1992; Wyse et al., 1994; Atkinson and Reilly, 1996; Edwards et al., 2007; Keisler et al., 2007) and it is possible that the ability to undergo changes in corticomotor excitability in response to repetitive motor training is also influenced by circadian rhythms. However, previous studies using plasticity inducing brain stimulation protocols would predict the opposite pattern of results than obtained in our present study (Sale et al., 2010). Future research is needed that objectively measures sleep quality by the use of electroencephalography (EEG) and experimentally modulates slow wave sleep which seems to be most related to synaptic downscaling and investigates whether, for example, slow wave sleep perturbation impacts on the renormalization of motor learning capacity. It is also important to note that we, tested two different groups of subjects. Even though, our groups were well matched regarding age, gender, over day activity and sleeping hours it might be advantageous to use a cross-over design in future studies.

## Conclusion

In this study, we show that the learning-induced synaptic plasticity caused by acquiring a finger sequence tapping task decreases after a day awake. Our findings are in line with the synaptic homeostasis hypothesis which states that synaptic strength is potentiated during the day and sleep restores learning capacity by maintaining synaptic potentiation within an optimal range. Hence, sleep ensures that M1 circuits can undergo reorganization to perform the practiced movements with high efficiency; a mechanism which is attenuated with time spent awake. Although our findings are in accordance with this hypothesis, future studies should objectively measure sleep quality and vary sleep independently of time of day to provide more direct evidence regarding the restorative role of sleep in synaptic homeostasis.

## Author Contributions

TTdeB designed the study; collected, analyzed and interpreted the data; drafted and revised the manuscript; gave final approval. JVS collected, analyzed and interpreted the data; drafted and revised the manuscript; gave final approval. RH interpreted the data; revised the manuscript; gave final approval. NW designed the study; interpreted the data; revised the manuscript; gave final approval.

## Conflict of Interest Statement

The authors declare that the research was conducted in the absence of any commercial or financial relationships that could be construed as a potential conflict of interest.
